# Molecular Characterization of the First Ebola Virus Isolated in Italy, from a Health Care Worker Repatriated from Sierra Leone

**DOI:** 10.1128/genomeA.00639-15

**Published:** 2015-06-18

**Authors:** Concetta Castilletti, Fabrizio Carletti, Cesare E. M. Gruber, Licia Bordi, Eleonora Lalle, Serena Quartu, Silvia Meschi, Daniele Lapa, Francesca Colavita, Roberta Chiappini, Antonio Mazzarelli, Patrizia Marsella, Nicola Petrosillo, Emanuele Nicastri, Giovanni Chillemi, Alessio Valentini, Alessandro Desideri, Antonino Di Caro, Giuseppe Ippolito, Maria R. Capobianchi

**Affiliations:** a National Institute for Infectious Diseases L. Spallanzani, Rome, Italy; b CINECA, Rome, Italy; c Department for Innovation in Biological, Agro-food and Forest Systems (DIBAF), University of Tuscia, Viterbo, Italy; d Molecular Digital Diagnostics (MDD), Viterbo, Italy; e Department of Biology, University of Rome Tor Vergata, Rome, Italy

## Abstract

Here, we report the complete genome sequence of an Ebola virus (EBOV) isolated from a health worker repatriated from Sierra Leone to Italy in November 2014. The sequence, clustering in clade 3 of the Sierra Leone sequences, was analyzed with respect to mutations possibly affecting diagnostic and therapeutic targets as well as virulence.

## GENOME ANNOUNCEMENT

Ebola virus (EBOV), causes a severe hemorrhagic disease in humans and nonhuman primates, with case fatality rates of 50% to 90% (1–3). In December 2013, EBOV emerged in western Africa in Guinea (4), spreading into Sierra Leone, Liberia, Senegal, Nigeria, and Mali. Western Africa is currently witnessing the most extensive Ebola virus outbreak so far recorded (26,593 reported cases, and 11,005 deaths as of 6 May 2015 [5]).

In an early study, Gire et al. observed in the EBOV variant causing the western Africa outbreak a substitution rate roughly twice as high as that in previous outbreaks (6). This raised concerns about possibly increased virulence and transmissibility of the virus (7) and failure of diagnostic tests predominantly based on reverse transcription (RT)-PCR (8). Moreover, high genetic variation could affect the efficacy of vaccines and therapeutic options, such as small interfering RNA (siRNA) (9) and phosphorodiamidate morpholino oligomers (PMOs) (10) or antibodies (i.e., ZMAPP, ZMAB, and MB-003) (11–16). More recently, a nucleotide substitution rate consistent with that observed in previous outbreaks has been inferred by Hoenen et al. based on additional full-length sequences from two clusters of infections imported to Mali in October and November 2014 (17).

We obtained the complete genome sequence of the virus isolate (Ebola virus/*H. sapiens*-tc/SLE/2014/Makona-Italy-INMI1) originating from a health worker evacuated from Sierra Leone to Italy in late November 2014. The virus was isolated on Vero E6 cells. Viral RNA was extracted (Roche) from the first passage; the complete genome was amplified in 45 overlapping fragments with EBOV-specific primers using the One-Step RT-PCR kit (Qiagen). The fragments were sequenced from both ends using Sanger techniques and assembled with the Sequencher software (GenCode).

The INMI1 isolate sequence shows high similarity with the previously published sequences from the western African outbreak, grouping in the phylogenetic maximum-likelihood tree within the Sierra Leone clade 3 (18). Single nucleotide variants (SNV) of the INMI1 isolate are located both in coding (mostly synonymous) and in noncoding regions. Eleven SNV are unique for the INMI1 isolate: 6 intergenic (t3008c, t3011c, t9659c, t10196c, a11111g, and a18728g), 1 synonymous in the VP24 gene (t10479a:I>I), 2 nonsynonymous (a16679g:K>R and c16895t:T>I), 1 synonymous (t15594c:D>D), and 1 synonymous back mutation (g11672a:L>L) in the L gene.

The INMI1 isolate sequence was aligned with routinely used primers and probe sets (19) to assess the potential impact of SNV on diagnostic PCR efficiency. Besides those already described, two unique variations were identified in the forward primer of a described RT-PCR targeting the L gene (20).

Compared to EBOV genomes used for drug design (21), some SNV (t17410c, c3890t, and c3902t) were identified in the siRNA target regions and none in the PMO target regions, and several amino acid mutations were identified in the antibody-binding sites (X47D, A82V, T262A, R314G, A315P, G331E, T336N, E359K, P382T, E405G, T411A, G440S, T441A, L443S, P446L, H455Y, A499T, and A503V).

No mutations potentially associated, at least in animal models, with increased pathogenicity (7) were identified.

### Nucleotide sequence accession number.

The complete genome sequence of the INMI1 isolate has been submitted to GenBank under the accession number KP701371.
